# MTAP loss correlates with an immunosuppressive profile in GBM and its substrate MTA stimulates alternative macrophage polarization

**DOI:** 10.1038/s41598-022-07697-0

**Published:** 2022-03-09

**Authors:** Landon J. Hansen, Rui Yang, Kristen Roso, Wenzhe Wang, Lee Chen, Qing Yang, Christopher J. Pirozzi, Yiping He

**Affiliations:** 1 The Preston Robert Tisch Brain Tumor Center, Duke University Medical Center, Durham, NC USA; 2 Department of Pathology, Duke University Medical Center, Durham, NC USA; 3 Department of Pharmacology and Cancer Biology, Duke University Medical Center, Durham, NC USA; 4 School of Nursing, Duke University Medical Center, Durham, NC USA; 5 Duke University Medical Center, 203 Research Drive, Medical Science Research Building 1, Room 159, Durham, NC 27710 USA; 6 Duke University Medical Center, 203 Research Drive, Medical Science Research Building 1, Room 199A, Durham, NC 27710 USA

**Keywords:** CNS cancer, Tumour immunology, Cancer microenvironment

## Abstract

Glioblastoma (GBM) is a lethal brain cancer known for its potent immunosuppressive effects. Loss of *Methylthioadenosine Phosphorylase* (*MTAP*) expression, via gene deletion or epigenetic silencing, is one of the most common alterations in GBM. Here we show that MTAP loss in GBM cells is correlated with differential expression of immune regulatory genes. In silico analysis of gene expression profiles in GBM samples revealed that low *MTAP* expression is correlated with an increased proportion of M2 macrophages. Using in vitro macrophage models, we found that methylthioadenosine (MTA), the metabolite that accumulates as a result of MTAP loss in GBM cells, promotes the immunosuppressive alternative activation (M2) of macrophages. We show that this effect of MTA on macrophages is independent of IL4/IL3 signaling, is mediated by the adenosine A_2B_ receptor, and can be pharmacologically reversed. This study suggests that MTAP loss in GBM cells may contribute to the immunosuppressive tumor microenvironment, and that *MTAP* status should be considered for characterizing GBM immune states and devising immunotherapy-based approaches for treating *MTAP*-null GBM.

## Introduction

Immunotherapy has become a mainstay of cancer treatment and has reshaped the way we understand and approach certain cancer types^1,2^. Despite recent progress, however, the promise of immunotherapy-based approaches for treating brain tumors, in particular high-grade glioblastoma (GBM), remains to be fully realized^3–5^. GBM is the most common and lethal brain tumor, with a dismal median survival of 12–15 months from the time of diagnosis^6^. It is also characterized by its profoundly immune-suppressive nature^7,8^. It has been well-established that GBM cells actively employ multiple strategies to escape immune surveillance and to create an immunosuppressive microenvironment^9,10^. As such, to fully harness the power of immunotherapy for GBM requires better understanding and more effective strategies for addressing the tumors’ immuno-suppressive countermeasures.

Recent genomic studies have provided insight into the molecular mechanisms of GBM pathogenesis, revealing many commonly mutated genes in tumor cells^11,12^. Further studies, in both glioma and other types of cancer, have drawn association between genetic alterations and tumor evasion of immune surveillance, providing rationale for tailoring treatments to the cancer cells’ genetic composition^13–15^. As an example, recent discoveries have linked *IDH1* mutations in glioma to immune evasion through interference of immune activation pathways, providing opportunities to develop highly specific immunological treatments^16–19^.

One of the most common genetic alterations in GBM, occurring in approximately 45% of all cases, is the homozygous deletion or epigenetic silencing of methylthioadenosine phosphorylase (*MTAP*)^11,20^. We have recently demonstrated that *MTAP* deletion is associated with increased tumorigenesis and with shortened disease-free survival in GBM patients^21^. MTAP is a metabolic enzyme that functions in the salvage pathway of adenine and methionine, and loss of MTAP results in the accumulation of its direct substrate, methylthioadenosine (MTA), which can be released into the tumor environment^22–25^. Aberrantly accumulated MTA is known to be functionally active within cells as an inhibitor of methyltransferases^23,24^, including interfering with intracellular protein methylation in T cells^26^, as well as acting on adenosine receptors on the cell surface of melanoma cell lines^27^. Studies of pathogen-induced host inflammatory responses have linked MTA to downregulation of TNFα production by macrophages through engaging adenosine receptors^28^, and have revealed a role of MTA in controlling the host inflammatory response^29^, such that MTA has been used as an immunosuppressive drug for treating colitis, liver inflammation, brain inflammation and autoimmunity in animal models^30–32^.

In this study, we investigated the link between MTAP loss in GBM cells and the GBM microenvironment. We reveal that in GBM tissues, low expression of MTAP (due to deletion or epigenetic silencing) is associated with immune cell populations indicative of an immunosuppressive state. We provide evidence that MTAP loss-induced MTA accumulation stimulates M2 alternative macrophage activation. We illustrate that this effect of MTA on macrophages is independent of interleukin (IL)-4/IL-13 signaling, is mediated by the adenosine A_2B_ receptor, requires STAT3, and is distinct from the actions of adenosine. Finally, we show that the MTA-induced alternative macrophage activation can be pharmacologically reversed. These results provide a basis for pursuing adenosine receptor signaling as a component of tumor-mediated immunosuppression and potential target in *MTAP*-null GBM.

## Results

### Loss of MTAP expression is associated with an immunosuppressive molecular profile in GBM

**Figure 1:**
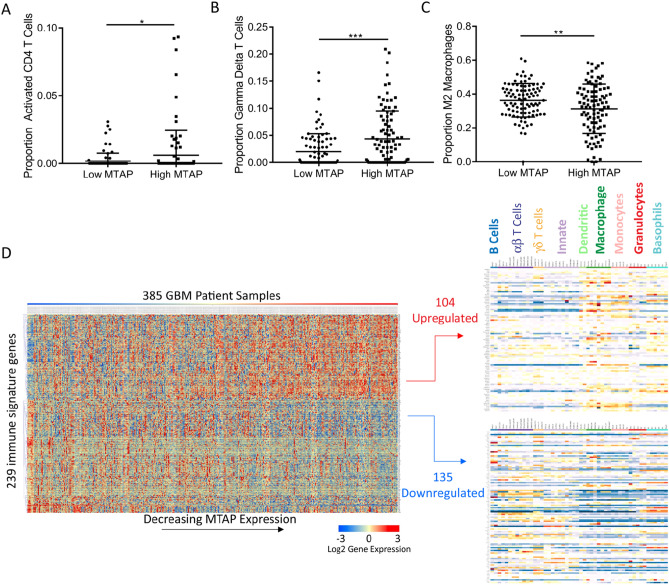
MTAP loss is correlated with an immunosuppressive gene signature in GBM samples. (**A**) Comparison of upper and lower *MTAP* expression quartiles (n = 96 each category) using CIBERSORT analysis of GBM gene expression microarray data shows lower activated CD4 T cells in tumors with low *MTAP* expression, *t* test *P* value < 0.03. (**B**) GBM samples with the lowest quartile *MTAP* expression have fewer gamma delta T cells, *t* test *P* value = 0.0002. (**C**) GBM samples with low *MTAP* expression have higher proportions of M2 macrophages, *t* test *P* value = 0.0064. (**D**) Heatmap showing expression of 239 immune signature genes that are differentially expressed between the lower and upper *MTAP* expression quartiles in GBM patients. The gene list for the heatmap was derived from 782 immune-specific genes identified by Charoentong et al. Only 641 of those 782 genes were measured in the TCGA GBM microarray data set. Of the 641 genes in the GBM dataset, 239 genes were expressed at significantly different levels (Benjamini–Hocbberg corrected t test *P* value < 0.0185) in samples with low MTAP expression compared to samples with high MTAP expression. All differentially expressed genes were included in the heatmap. In order to classify the upregulated and downregulated genes identified, each clustered gene set was analyzed using data from the immunologic genome project (https://www.immgen.org), which uses a compendium of microarray data (366 microarrays/37 studies) from specific immune cell types to identify which immune cells express the genes of interest. Our analysis revealed two unique expression patterns. Genes expressed at higher levels in the low-MTAP samples are more representative of innate immune cells such as macrophages and monocytes, while genes downregulated in low-MTAP cells are more representative of activated CD4 T cells and innate lymphocytes.

To explore the association of *MTAP* expression with the immune cell profile in GBM we employed CIBERSORT, a method for deconvoluting gene expression data and estimating immune cell fractions in human cancers^33–35^. This method has been successfully used for analyzing a large number of samples across multiple cancer types^34^, is readily compatible with the microarray-based platform of the largest available GBM expression dataset, and gives unique consideration to data normalization and noise control, which is essential when analyzing heterogeneous and diffusive tumors, such as GBM^36^. We applied this analysis to the GBM dataset from TCGA (385 GBM patient samples)^11^ to examine the link between MTAP loss and the GBM immune microenvironment. As MTAP alteration can occur through homozygous gene deletion or epigenetic silencing, we compared the two groups of patients with the highest and lowest *MTAP* expression (upper and lower quartiles, n = 96 each) (Supplementary Fig. S1). This revealed reduced fractions of activated CD4 T cells and increased resting CD4 T cells in tumors with low *MTAP* expression (Fig. 1A, Supplementary Fig. S1). Additionally, two types of innate immune cells were identified as differentially represented between these two groups of tumors. There was a lower fraction of γδT cells (Fig. 1B), and a significantly higher fraction of M2 macrophages in the low-*MTAP* expressing tumors (Fig. 1C).

To corroborate the findings from CIBERSORT analysis, we utilized a second recently developed platform for identifying individual immune cell types, which is based on a dataset of 366 microarrays compiled from multiple independent studies^37^. This data set was previously used to generate a list of 782 genes that are specific to immune cell subpopulations (i.e. not expressed in tumor cells or normal tissue)^37^. We analyzed this list of genes in the TCGA GBM dataset and found that among the 641 of these genes for which expression data was available, 239 of them were differentially expressed between the MTAP-low and MTAP-high GBM populations (upper quartile vs lower quartile, n = 96 each group, Benjamini–Hochberg corrected p-value < 0.0185). Hierarchical clustering revealed two groups of genes that could be evaluated to compare immune cell types upregulated and downregulated in MTAP deficient GBM (104 genes upregulated in MTAP-low tumors, 135 genes downregulated in MTAP-low tumors) (Fig. 1D; Supplementary Table 1). Using a database compiled by the Immunologic Genome Project^37^, we found that the genes upregulated in low-MTAP tumors were predominantly associated with macrophages and monocytes, while those genes downregulated in low-MTAP tumors represent activated CD4 T cells and innate lymphocytes (Fig. 1D; Supplementary Figs. S2–S4). Collectively, the findings from various analyses of the gene expression profiles in GBM samples with low versus high MTAP expression suggest a reduction in immune-reactive T cells^38^ and an increase in immunosuppressive M2 macrophages^39^ in samples with low *MTAP* expression, opening the possibility that MTAP deficiency is linked to the immunosuppressive GBM microenvironment. Of note, CIBERSORT and Immunologic Genomic project analyses revealed differences in expression of several immune cell types including plasma cells, memory B cells, NK cells, and dendritic cells. While all these differences in the tumor immune profile may be linked or regulated by a similar mechanism and warrant additional investigation, further work presented in this study focuses on macrophage regulation as a model for MTAP deficiency regulating the immune microenvironment.

### MTA stimulates alternative activation of macrophages

Macrophages are the most abundant immune cell type in GBM, representing as many as half of all cells in the tumor mass^40^, where they are known to play an immunosuppressive/pro-tumor role^39,41^. Macrophages are traditionally classified as M1 (inflammatory) or M2 (anti-inflammatory). M2 macrophages are classically stimulated by cytokines IL-4, IL-13, IL-10, and M-CSF and are associated with reduction of inflammatory signals, resolution of the immune response, wound healing, and tumor promotion^42,43^. Higher numbers of M2 tumor-associated macrophages have been linked to poor cancer prognosis^43,44^. Upregulated numbers of M2 macrophages in MTAP deficient GBM samples from our data analysis suggests a more immunosuppressive environment with reduction of inflammatory signals and decreased antigen presentation, potentially contributing to the decreased numbers of activated CD4 lymphocytes observed in these samples as well.

Macrophages can be regulated by adenosine signaling^45^, which potentiates the effect of cytokines in promoting alternative macrophage activation through adenosine A_2_ receptors^46^. This led us to hypothesize that the MTAP substrate, MTA, an adenosine analogue that is secreted by *MTAP* deleted cells, impacts macrophage polarization through adenosine receptor signaling. In fact, studies focusing on pathogenic mechanism and treatment of pathogen-induced host inflammatory responses have found that MTA can suppress lipopolysaccharide (LPS)-induced expression of inflammatory response genes, such as *TNF*, via adenosine A_2_ receptors^28,47^.

**Figure 2:**
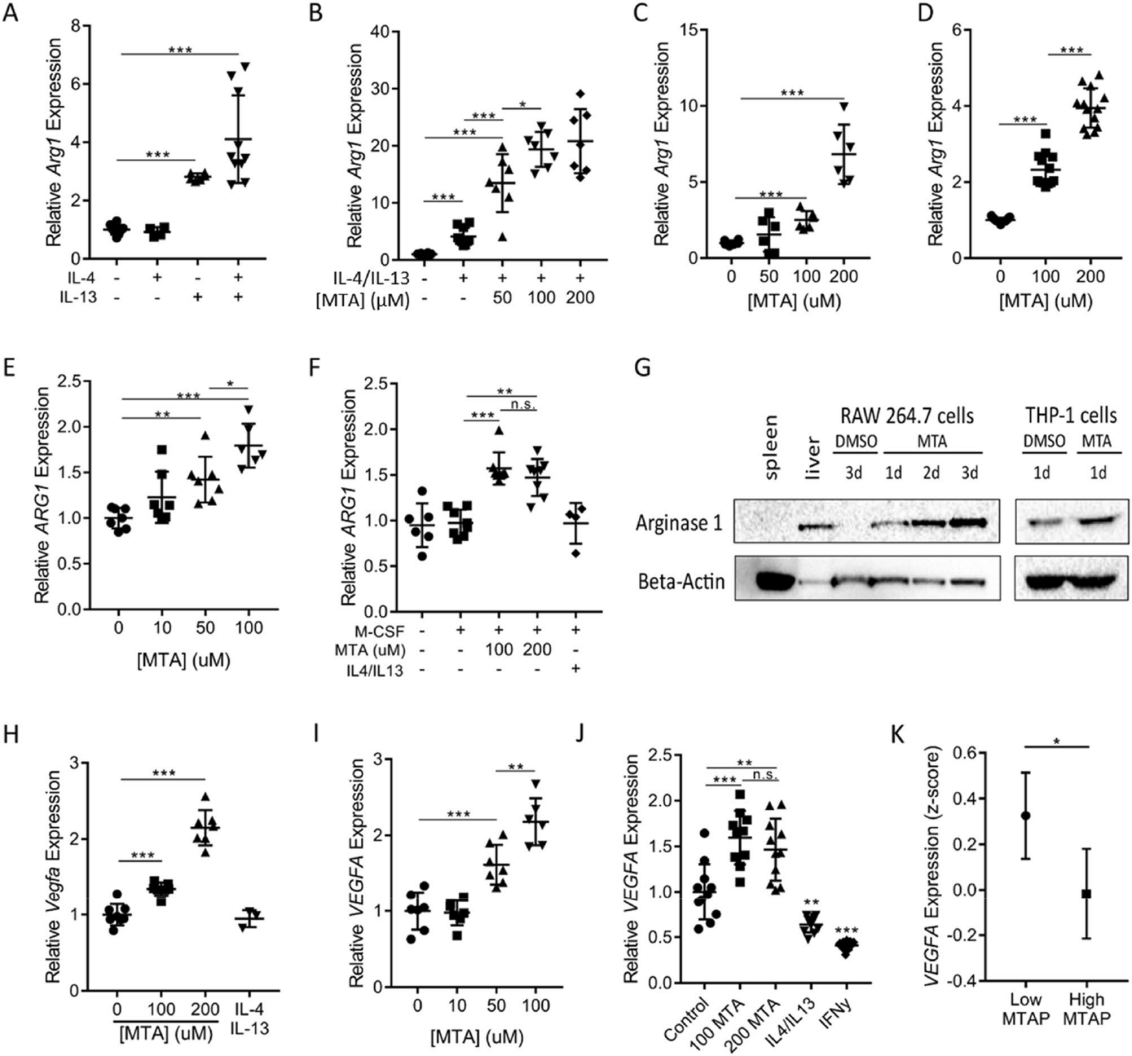
MTA promotes upregulation of M2 macrophage marker genes. (**A**) RAW 264.7 cells were treated with IL-4 and IL-13 individually or in combination (5 ng/mL for each cytokine) for 12 h before total RNA was prepared for *Arg1* expression analysis (all gene expression was measured by RT-qPCR, n ≥ 5). (**B**) RAW 264.7 cells were treated with the indicated cytokines as in (A), together with different doses of MTA, after which relative *Arg1* expression was determined as above, n ≥ 5. (**C**) RAW 264.7, (**D**) BV-2, and (**E**) THP-1 cells were treated with different doses of MTA for 12 h and *Arg1* expression was measured, n ≥ 5. (**F**) Primary human monocytes were differentiated to macrophages with M-CSF and treated with MTA or IL-4/IL-13 for 24 h and *Arg1* expression levels were measured. Though the impact of cytokines on these cells was less robust than on RAW 264.7 cells, it is notable that MTA treatment still resulted in measurable differentiation while IL-4/IL-13 did not. (**G**) Immunoblots confirmed the MTA-stimulated expression of ARG1; “d” denotes “days”. (**H**) RAW 264.7, (**I**) THP-1, and (**J**) primary human monocyte cells were treated with MTA for 12 h, and total RNA was isolated for measuring *VEGFA* expression, n ≥ 4. In Fig. (J): “100” denotes “100 uM”, and “200” denotes “200 uM”. (**K**) TCGA human GBM microarray data were used for analyzing *VEGFA* expression in tumors with low or high *MTAP* expression, n = 96 patients per group. All experiments were repeated independently. All statistical comparisons were performed using ANOVA for group comparisons or unpaired student’s t-test for individual comparisons; error bars indicate mean ± SD; *P < 0.05, **P < 0.005, ***P < 5 × 10^–4^, n.s. = not significant.

To investigate the impact of MTA on macrophage activation, we utilized the well-established murine RAW 264.7 macrophage cell line model^46,48^. As a control, RAW 264.7 cells were treated with M2 macrophage-inducing Th2-type cytokines, IL-4 and IL-13, which drove the cells toward alternative (M2) activation as expected, demonstrated by the upregulated expression of *Arginase 1* (*Arg1*), a canonical marker for M2 macrophages^49^ (Fig. 2A). When MTA was added to the IL-4/IL-13 treatment, induction of *Arg1* expression was dramatically augmented (Fig. 2B). Remarkably, when MTA was used as a single agent to treat the cells, upregulated expression of *Arg1* was also observed (Fig. 2C). This upregulation of *Arg1* transcription was not limited to RAW 264.7 cells as it was also observed in the murine BV-2 microglial cell line, the human THP-1 monocytic cell line, and in primary human monocytes (Fig. 2D–F). This rise in transcription was followed by a rise in Arginase-1 protein levels (Fig. 2G). Notably, despite similarly upregulating *Arg1* expression, the overall effect of MTA on macrophages was distinct from that of IL-4/IL-13, as treatment of cells with MTA attenuated the IL-4/IL-13-induced expression *Pparg* and *Mrc1*, marker genes associated with the M2a macrophage subtype (Supplementary Fig. S5A,B)^50,51^. Instead, MTA activated the expression of *Vegfa*, a classic feature of the M2d subtype and tumor associated macrophages (TAMs) (Fig. 2H)^51^. This phenotype was validated in human THP-1 cells and primary human monocytes differentiated into macrophage lineage (Fig. 2I,J). Prompted by this finding, we revisited the GBM gene expression data (TCGA) and confirmed that in GBM patients *VEGFA* expression was significantly higher in samples with low MTAP expression (Fig. 2K). Collectively, these results suggest that MTA promotes alternative macrophage activation resembling the M2d subtype and that this process is likely occurring in MTAP deficient GBMs.

To validate that the phenotype induced by exogenously administered MTA is relevant to the amount of MTA produced by tumor cells, we generated an isogenic *MTAP*-null derivative of the murine GBM cell line CT-2A. As expected, CRISPR-mediated homozygous deletion of *Mtap* in CT-2A cells led to the accumulation of MTA in the culture media (Supplementary Fig. S5C). We then tested the response of macrophages to spent media from the *Mtap*-null cell line as a way of simulating the tumor microenvironment. Exposure of RAW 264.7 cells to spent media from *Mtap*-null CT-2A cells indeed induced *Arg1* expression, while exposure to the spent media from parental CT-2A (*Mtap* wildtype) cells had a minimal effect on *Arg1* expression and the addition of exogenous MTA to the conditioned media was able to stimulate an alternative activation response (Supplementary Fig. S5D). Together, these results support the hypothesis that MTA in the extracellular compartment of *MTAP*-null GBM cells can work in concert with known M2 macrophage-stimulating cytokines in altering tumor associated macrophages, and/or directly promoting the alternative activation of macrophages as a single agent.

### Regulation of macrophage activation by MTA requires the adenosine A_2B_ receptor and STAT3

**Figure 3:**
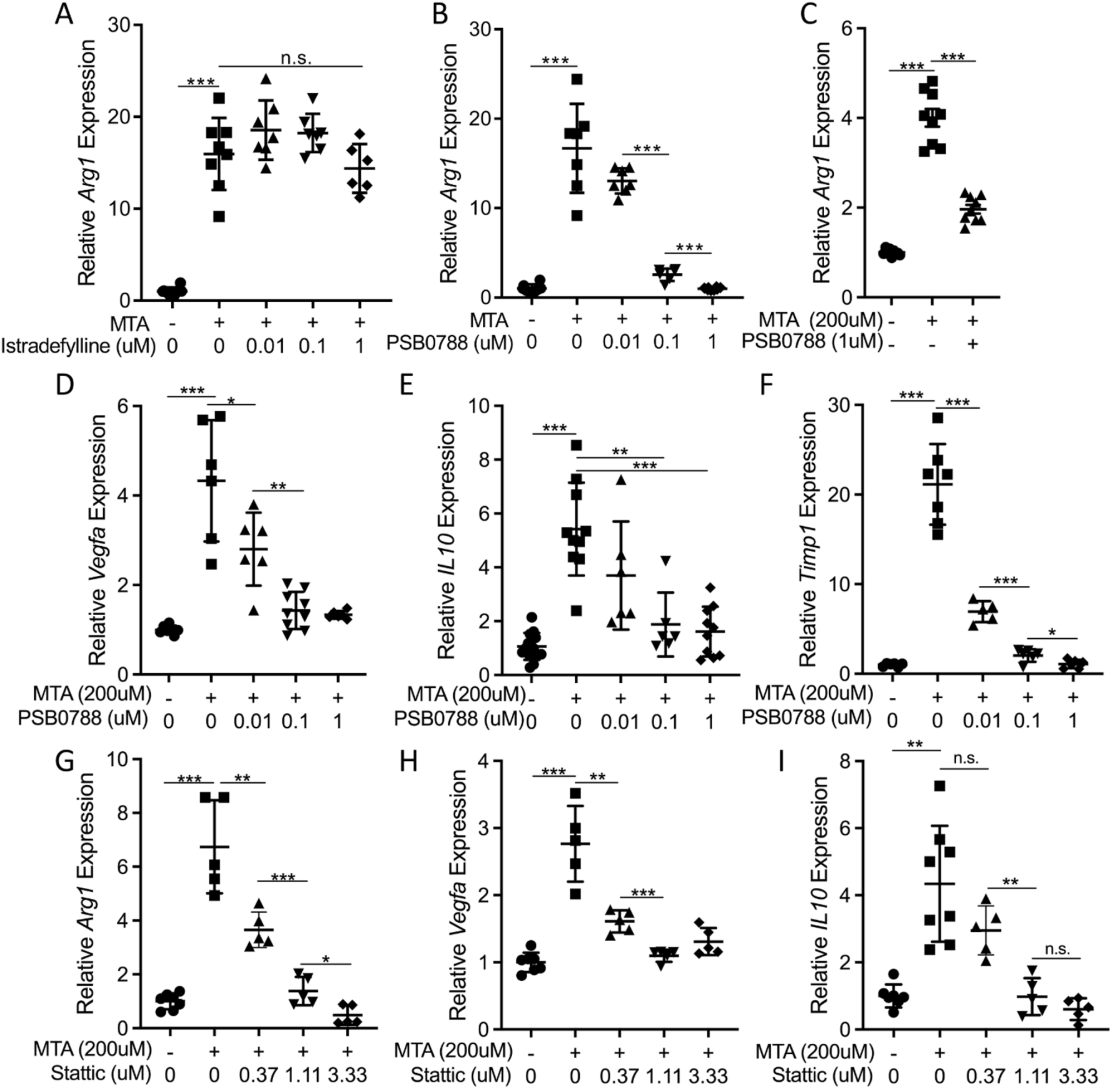
The impact of MTA on M2 macrophage marker genes is blocked by inhibition of the A_2B_ adenosine receptor and STAT3. (**A**,**B**) RAW 264.7 cells were treated with MTA for 12 h and simultaneously with (**A**) A_2A_ receptor antagonist Istradefylline or (**B**) A_2B_ receptor antagonist PSB 0788 and expression of *Arg1* was determined by RT-qPCR, error bars indicate mean ± SEM, n ≥ 5. (**C**) The BV-2 microglial cell line was treated with MTA with or without A_2B_ receptor antagonist PSB 0788 for 12 h and *Arg1* expression was measured by RT-qPCR, error bars indicate mean ± SEM, n ≥ 5. (**D**–**F**) RAW 264.7 cells were treated with MTA, with or without A_2B_ receptor antagonist PSB 0788 for 12 h and expression of (**D**) *Vegfa*, (**E**) *IL10* and (**F**) *Timp1* were determined by RT-qPCR, error bars indicate mean ± SEM, n ≥ 5. (**G–I**) RAW 264.7 cells were treated with MTA, with or without STAT3 inhibitor Stattic for 12 h, and expression of (**G**) *Arg1*, (**H**) *Vegfa*, and (**I**) *IL10* were determined by RT-qPCR, error bars indicate mean ± SEM, n ≥ 5. All statistical comparisons were done using ANOVA for group comparisons or unpaired student’s t-test for individual comparisons; *P < 0.05, **P < 0.005, ***P < 5 × 10^–4^, n.s. = not significant.

Previous work has shown that the action of adenosine in modulating the innate immune response to cytokine signaling is achieved through adenosine A_2_ receptors^51^. We tested whether the effects of MTA are also mediated through these receptors using specific antagonists of A_2A_ and A_2B_ receptors. We found that an A_2A_ receptor antagonist, Istradefylline, had only minimal effect on MTA-induced *Arg1* expression (Fig. 3A). In contrast, an antagonist of the A_2B_ receptor, PSB 0788, potently attenuated the expression of *Arg1* induced by MTA (Fig. 3B,C). Furthermore, PSB 0788 also blocked the induction of *Arg1* expression by the spent media of *MTAP*-null GBM cells (Supplementary Fig. S5D), and attenuated other MTA-stimulated M2 marker genes, including *Vegfa*, *Timp1*, and *IL10*, while countering the inhibitory effect of MTA on *Mrc1* expression (Fig. 3D–F; Supplementary Fig. S5B)^46,48^. The A_2A_ receptor inhibitor, Istradefylline, again had negligible effects on any of these target genes’ transcription in response to MTA (Supplementary Fig. S6A–C). In order to rule out potential off-target effects of the pharmacologic inhibitor and confirm involvement of the A_2B_ receptor in the phenomena we observed, we used CRISPR/CAS9 to genetically knock out the A_2B_ receptor gene, *Adora2b* (Supplementary Fig. S7). We saw decreased basal expression levels of *Arg1* and *Timp1* and a greatly dampened response to MTA (Supplementary Fig. S6D,E). Collectively, these results suggest that MTA acts through the adenosine A_2B_ receptor and that this receptor is involved in regulating macrophage activation states.

We next asked whether a sustained contribution of the A_2B_ receptor signaling pathway is required for maintaining the alternative macrophage activation state. To address this question, we treated the cells with the A_2B_ receptor antagonist at a delayed time point following treatment with MTA. We found that treatment of the already activated macrophages with the adenosine A_2B_ receptor antagonist was still able to abolish *Arg1* expression (Supplementary Fig. S5E), suggesting the effect of MTA can be pharmacologically reversed, an important point for determining any translational therapeutic potential.

To illuminate the downstream mediators of MTA-stimulated A_2B_ receptor signaling, we tested the role of transcriptional regulators CREB and STAT3, as these are reported to function downstream of adenosine receptors and are known to regulate *Arg1*, *Vegfa*, and *IL10*, among other genes^52–56^. We found that the inhibition of STAT3 completely abrogated the MTA-induced expression of all three genes. In contrast, inhibition of CREB had only marginal or no effect on MTA-induced gene expression (Fig. 3G–I; Supplementary Fig. S8A–C). We then utilized a dominant-negative construct of STAT3 to genetically reduce STAT3 activity and again observed decreased response to MTA (Supplementary Fig. S8D)^57^. Interestingly, we were not able to reliably measure any change in STAT3 phosphorylation (p-Tyr705) levels in response to MTA at the time points measured (Supplementary Fig. S8E), suggesting such a phosphorylation change was not necessary for MTA-stimulated A_2B_ receptor signaling in potentiating alternative macrophage polarization. This finding is consistent with known roles of STAT3 in regulating gene expression and chromatin organization, and as an important component of cancer and immune biology as previously demonstrated^58–61^.

### MTA and adenosine activate distinct yet overlapping signaling pathways

**Figure 4:**
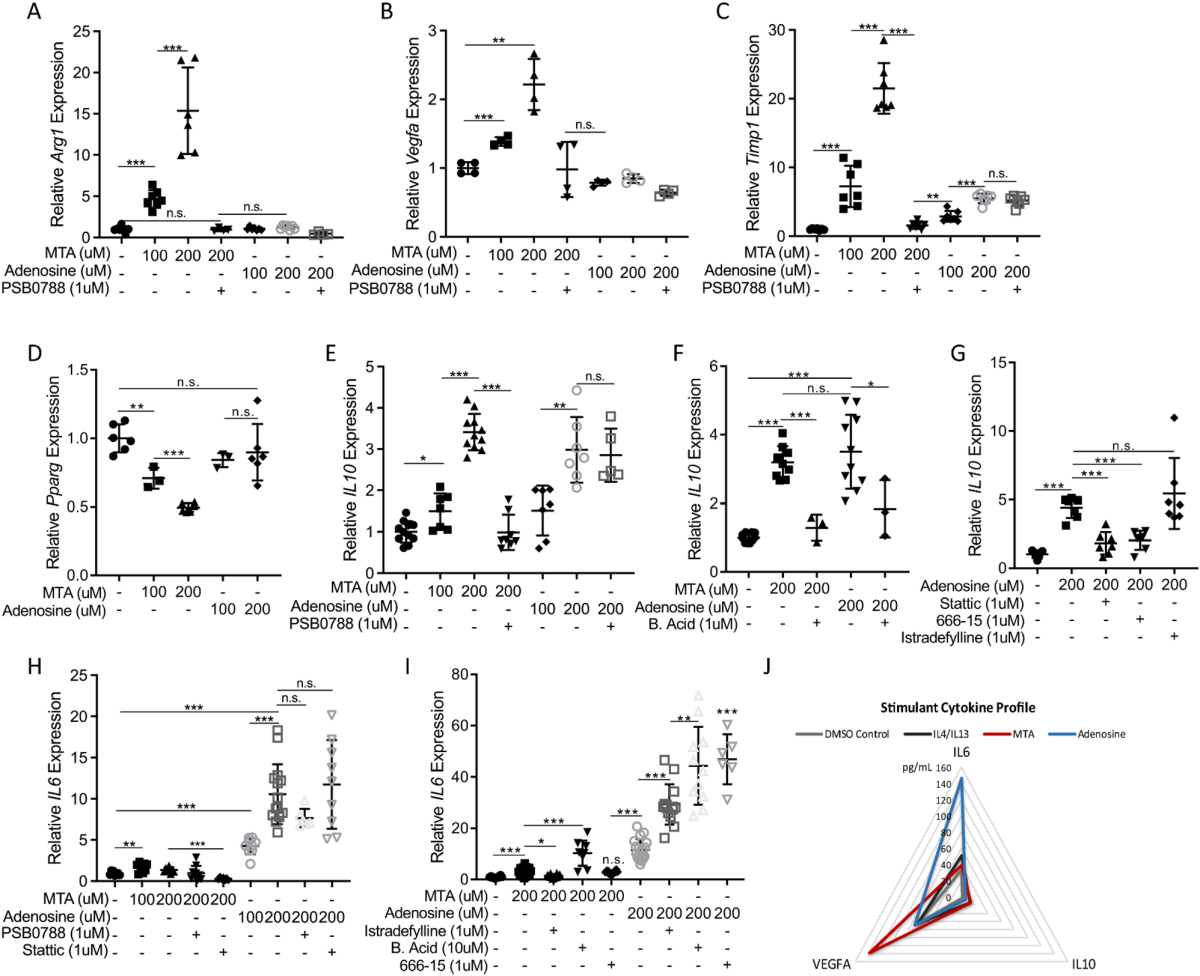
Distinct MTA and adenosine signaling. (**A**–**C**) RAW 264.7 cells were treated with the MTA or adenosine with or without A_2B_ receptor antagonist PSB 0788 for 12 h and (**A**) *Arg1*, (**B**) *Vegfa*, and (**C**) *Timp1* expression were measured by RT-qPCR, error bars indicate mean ± SD, n ≥ 4. (**D**) RAW 264.7 cells were treated with MTA or Adenosine for 12 h and *Pparg* expression was measured by RT-qPCR, n ≥ 5. (**E**,**F**) Raw 264.7 cells were treated with MTA or adenosine for 12 h with or without (**E**) A_2B_ receptor antagonist PSB 0788 or (**F**) C/EBP inhibitor betulinic acid (B. Acid) and *IL10* expression was measured by RT-qPCR, n ≥ 5. (**G**) *IL10* expression was measured by RT qPCR following adenosine treatment with or without STAT3 inhibition (Stattic), CREB inhibition (666-15), or A_2A_R antagonist Istradefylline, n ≥ 5. (**H**,**I**) RAW 264.7 cells were treated with MTA or adenosine for 12 h with or without the indicated inhibitors and *IL6* expression was measured by RT-qPCR, n ≥ 5. (**J**) RAW 264.7 cells were treated with the indicated components, denoted by color codes, and cytokines in spent media were measured using Luminex technology after 2 (IL-6) or 3 (IL-10, VEGFA) days. The diagram shows the concentrations of each cytokine in the media. All statistical comparisons were performed using ANOVA for group comparisons or unpaired student’s t-test for individual comparisons; Error bars indicate mean ± SD; *P < 0.05, **P < 0.005, ***P < 5 × 10^–4^, n.s. = not significant.

As both adenosine and MTA signal through adenosine receptors, we sought to determine if there were any quantitative or qualitative differences in their effect on macrophage activation. We treated RAW 264.7 macrophages with equimolar concentrations of adenosine or MTA and tested the expression of macrophage activation markers in response to each metabolite. To our surprise, we found that MTA much more potently stimulated expression of *Arg1*, *Vegfa*, and *Timp1* than did adenosine (Fig. 4A–C). In addition, MTA was unique in its ability to suppress *Pparg* expression (Fig. 4D). Furthermore, while MTA and adenosine similarly upregulated *IL10* expression (Fig. 4E), the impact of adenosine on *IL10* (and *Timp1*) was impervious to PSB0788, suggesting adenosine was acting through a different mechanism to induce expression of these genes.

We then tested the role of downstream mediators in producing the response to MTA and adenosine signaling. We found the effect of MTA and adenosine on *IL10* expression were both effectively blocked by betulinic acid, an inhibitor of transcription factor C/EBP (CEBPA/CEBPB) (Fig. 4F), consistent with reports of C/EBP being a critical regulator of *IL10* expression^62^ and indicating a convergence of the adenosine and MTA signaling pathways through this transcription factor. Furthermore, similar to what we had observed with MTA (Fig. 3G), induction of *IL10* expression by adenosine was also blocked by stattic, indicating adenosine-stimulated *IL10* expression requires STAT3 (Fig. 4G). However, whereas MTA-induced expression of *IL10* was independent of CREB (Supplementary Fig. S8B), addition of CREB inhibitor 666-15 effectively blocked *IL10* expression in response to adenosine (Fig. 4G), indicating that in the downstream signaling pathways there are distinct mechanisms responsible for MTA and adenosine-mediated responses in macrophages.

To further illuminate the distinct/overlapping roles of MTA and adenosine, we investigated the expression of *IL6*, another important regulator of macrophage activation^63,64^ that has been reported to be upregulated in response to adenosine signaling^65–69^. We found that adenosine much more potently upregulated *IL6* than did MTA, and that while inhibition of STAT3 decreased *IL6* expression in the context of MTA treatment, STAT3 inhibition had no significant effect on *IL6* expression following adenosine treatment (Fig. 4H). Furthermore, adenosine-mediated upregulation of *IL6* expression was further potentiated by A_2A_ receptor antagonist Istradefylline, as well as by inhibition of CREB or C/EBP, while the effect of MTA was only potentiated by the C/EBP inhibition, not CREB or A_2A_ receptor blockade, suggesting there are different negative feedback signaling pathways for MTA and adenosine (Fig. 4I). Thus, while MTA and adenosine both upregulate *IL6* expression (adenosine to a greater extent), the dramatic differences in *IL6* regulation following MTA or adenosine stimulation further demonstrate the distinctive receptors and signaling pathways activated by these two metabolites.

To confirm the cytokine production measured by gene expression analysis, we utilized multiplex cytokine quantification to measure IL-6, IL-10, and VEGFA protein levels in spent media from RAW 264.7 cells treated with MTA or adenosine. This analysis of cell protein production confirmed the gene expression findings detailed above, with increased VEGFA and IL-10 levels in response to MTA exposure and clear IL-6 and IL-10 production following adenosine treatment (Fig. 4J; Supplementary Fig. S9).

Collectively these results suggest that MTA and adenosine each engage a distinct balance of adenosine receptor signaling, resulting in divergent pathway activation, gene expression profiles, and cytokine production. The unique effect of MTA on macrophages, which is not shared by adenosine and requires the adenosine A_2B_ receptor, STAT3, and C/EBP, supports an immunosuppressive, M2-polarizing effect of aberrant MTA accumulation on macrophages in MTAP-deficient GBM.

## Discussion

Understanding and targeting the immuno-suppressive mechanisms of GBM is a critical step toward improving treatment for this lethal cancer type^9^. In this study, we provide multiple lines of evidence to link MTAP loss, one of the most common genetic/epigenetic events in GBM, to changes in the tumor immune microenvironment. We demonstrate using patient data that *MTAP* deleted GBMs contain an immunosuppressive gene expression signature, with increased numbers of M2 macrophages and decreased lymphocyte activation. Our findings resonate with a recent study showing that loss of the *CDKN2A*/*MTAP* locus confers an immunosuppressive microenvironment in human cancers^70^, raising the possibility that MTAP loss is an underlying factor. Utilizing in vitro macrophage models, we verified that the process of M2 polarization is influenced by MTA signaling through the adenosine A_2B_ receptor, resulting in upregulation of M2 marker genes *Arg1*, *IL10*, and *Vegfa*. This represents a unique mechanism of alternative macrophage activation and a potentially significant contribution to the immunosuppressive tumor microenvironment in GBM, which is known for an abundance of immunosuppressive tumor-associated macrophages^71^. It has previously been demonstrated that MTA can directly suppress the proliferation and function of T lymphocytes^26,72^. Our findings complement these studies by showing that MTA can also influence innate immune cells.

The finding that the macrophage response to MTA is distinct from the response to adenosine suggests that MTAP-deficient GBMs with aberrant MTA accumulation likely have an impact on the immune environment in a manner that is qualitatively and/or quantitatively different from tumors which simply accumulate adenosine as a mechanism of immune evasion^73,74^. The role of adenosine receptor signaling in regulating inflammation, as well as its tumor-promoting functions, are beginning to be more widely recognized^75^. Adenosine receptors and the adenosine-generating enzymes, CD39 and CD73, are being investigated as therapeutic targets, either through stimulating or countering the adenosine receptor signaling pathways to control pathogenic inflammation or treat advanced cancers^76–78^. In GBM, adenosine (and upregulation of CD39 and CD73) has been shown to contribute to tumor-mediated immunosuppression^79^. Additionally, A_2B_ receptor signaling has been shown to promote tumor metastasis and stimulate tumor angiogenesis^80,81^. Our finding that the effect of MTA on macrophage functionality is mediated by the adenosine A_2B_ receptor and is amenable to pharmacological intervention adds a new line of evidence to support the rationale of targeting A_2B_ receptor signaling in MTAP-deficient GBM. Development of MTAP-deficient, immune-competent GBM models will be necessary for further investigating the effectiveness of this strategy in vivo. We speculate that given the abundance of tumor-associated macrophages and the characteristic immunosuppressive microenvironment of GBM, altering MTA-mediated purinergic signaling has the potential to significantly impact tumor growth and augment immunotherapeutic interventions in *MTAP*-deleted GBM.

We note that experimental findings from the in vitro cell line models bear major limitations and may not fully explain the observed link between immunosuppression and MTAP status. In addition, those experimental results represent only a limited view of the complex time scale of the signaling mechanisms and responses involved. We suggest that further research utilizing human GBM specimens and in vivo, immunocompetent orthotopic GBM models will be necessary to assess the functional interplay between MTAP loss and the innate immune cells in GBM, and how they collectively influence the adaptive immune characteristics within the tumor.

## Materials and methods

### Cell lines and cell culture

RAW 264.7 and THP-1 macrophage cell lines were obtained from the Duke Cell culture Facility. BV-2 cells were a generous gift from Dr. Tso-Pang Yao. RAW 264.7 and BV-2 cells were maintained in DMEM with 4.5 g/L l-glucose (Sigma Cat #D6429) supplemented with 10% heat-inactivated FBS and anti-anti (antibiotic/antimycotic). THP-1 cells were cultured in RPMI 1640 with 10% heat-inactivated FBS and anti-anti. CT-2A cells were a generous donation from Dr. Darrell Bigner. The cells were maintained in DMEM/F12 (Gibco Cat #11330-032) supplemented with B-27 (Gibco Cat #17504-044), EGF (Stemcell), and FGF (Stemcell) and grown in suspension. Primary tissue cultures were derived with consent from patient tumor samples obtained by the Duke Brain Tumor Center. These patient-derived cultures were maintained in human neural stem cell (NSC) media (STEMCELL, cat# 05751), supplemented with EGF, FGF, and Heparin and plated onto laminin coated plates. All experiments were performed within the first 20 passages.

### Plasmid construction and generation of derivative cell populations

The CRISPR system was used for knockout of *Mtap* in CT-2A. Two double-stranded oligonucleotides that encode sgRNA targeting exon 1 (CCTCGGGCTCCGCCTGCACGGCG) and exon 3 (CCATCCGATGCCTTAATTTTGGG) of *Mtap* were cloned into the px552-pEASY plasmid. CT-2A cells were transfected simultaneously with sgRNA plasmid and the px458 plasmid containing cas9 and GFP. For transient plasmid transfection, plasmids (2 plasmids at 1:1 ratio for achieving the desired gene deletion/mutations) and Transfex (ATCC, cat# ACS-4005) were mixed and used for cell transfection according to manufacturer’s instructions. Three to four days after the transfection, green fluorescent protein–positive (GFP+) cells were sorted via fluorescence-activated cell sorting (BD FACSVantage SE cell sorter, Duke Cancer Institute) to obtain the GFP+ population. Sorted cells were plated at single-cell densities and allowed to expand for 21 days, at which point DNA was prepped from each colony to screen for a deletion in *Mtap* (exon 1–exon 3) using PCR amplification across the deleted region. RAW264.7 cell lines with A_2b_R gene knockout (ko) was obtained by CRISPR using sgRNA#1 (GACGCAAGACGCGCTGTACG) or sgRNA#2 (GGCCACGTCTGCCGTCGCCA), delivered by lentivirus construct LentiVRISPR-E (Addgene #78852). Transduced cells were selected with puromycin and resistant populations were genotyped by Sanger sequencing. For generating RAW264.7- STAT3-DN derivative line, RAW264.7 cells were transduced with lentivirus (Addgene, plasmid # 24984, EF.STAT3DN.Ubc.GFP, expressing dominant negative STAT3 and GFP), and GFP + cells were sorted for further experiments. sgRNA and primer sequences are shown in Supplementary Table 1.

### In vitro macrophage polarization

Raw 264.7 cells were plated in a 12 well dish and stimulated with methylthioadenosine (MTA) (Cayman Cat #15593) or adenosine (Sigma Cat #A9251) for 12 h, at which point they were collected for analysis. Cytokines IL4 (Peprotech Cat #200-04) and IL-13 (Peprotech Cat #200-13) were added within one hour of MTA/adenosine administration at a dose of 5 ng/mL. THP-1 stimulation with MTA was identical to RAW 264.7 cells except that the cells were first differentiated using PMA (Cayman Cat #10008014) for 24 h, then cultured in fresh media for 24 h prior to stimulation. BV2 cells were cultured in the same conditions as RAW 264.7 cells. Primary human monocytes were obtained frozen from STEMCELL Technologies (Cat # 70034). Cells were cultured in macrophage differentiation medium (STEMCELL Technologies, Cat # 10961) supplemented with M-CSF (STEMCELL, Cat # 78057.1) for 48 h, after which MTA was added and cells were harvested after 24 h. To test the impact of A_2B_ and A_2A_ receptors on MTA-mediated macrophage polarization, A_2A_R inhibitor Istradefylline (Selleckchem Cat #S2790) or A_2B_R inhibitor PSB 0788 (Tocris Cat #3199) were added simultaneously with MTA or adenosine, except in the indicated experiments where delayed administration of the A_2B_R inhibitor was tested. To further explore downstream signaling pathways STAT3 inhibitor Stattic (Tocris Cat # 2798), CREB inhibitor 666-15 (Tocris, Cat #5661) and C/EBP inhibitor betulinic acid (Tocris Cat #3906) were added at the time of MTA/adenosine administration. To test the effect of physiological accumulations of MTA from tumor cells, spent media from CT-2A parental or MTAP knockout cells was collected and added to the macrophage media in a 2:1 ratio and cells were plated for 12 h prior to measuring response.

### Preparation of RNA and RT- qPCR

Total RNA was extracted using quick-RNA mini prep kit (Zymo Research, cat# 11-328) following the manufacturer’s protocols. Concentration of RNA was determined by Nanodrop Lite Spectrophotometer (Thermo Scientific). For gene expression analysis, reverse transcription was performed to convert total RNA into complementary DNA (cDNA) using the RNA to cDNA EcoDry Premix (Clontech, cat #639547). Subsequently, real-time qPCR was performed following the aforementioned qPCR procedure. Each reaction included a cDNA template equivalent of 10 ng of total RNA. The glyceraldehyde 3-phosphate dehydrogenase (*GAPDH*) and beta-Actin genes were used as internal expression controls for RT-qPCR with reliable results. When beta Actin was used as the control amplicon the following program was followed: 95 °C, 3 min; 41 cycles of 95 °C 10 s and 68 °C 20 s, then a standard dissociation curve from 65 to 95 °C of 5 s/5° increment. When Gapdh was utilized as the internal control the following program was used: 95 °C, 3 min; 40 cycles of 95 °C 10 s, 60 °C 20 s, and 72 °C 1 s, then a standard dissociation curve from 65 to 95 °C of 5 s/5° increment. Relative quantification of expression was performed by comparing the Ct of target amplicons to the Ct of internal controls (GAPDH, beta-Actin) using the 2^−ΔΔCt^ method.

### Oligos and primers

All oligos and primers used for the study were synthesized by Eton Bio and are listed in Supplementary Table 1. Quantitative PCR was performed using KAPA SYBR Fast 2 × Universal master mix (KK4602) according to the manufacturer’s protocols on a BIO-RAD CFX96 Real-Time System.

### Western Blot

Arginase1 antibody was obtained from Thermofisher (Cat # 711765). Cells were treated with MTA for 24 h and cells were lysed with RIPA buffer and ran on a Bio-Rad ready-made gel and transferred to nitrocellulose membrane. Total STAT3 and p-Tyr705 STAT3 were obtained from Cell Signaling Technologies (Cat # 9139, 9145).

### Milliplex/luminex assays

To quantify cytokine levels in cell culture media, we utilized the multiplex cytokine quantification technology available from Milliplex. Custom kits were ordered that measure IL-6, IL-10, and VEGFA. Samples were processed according to manufacturer’s instructions and read on a Luminex machine in the Immunology Unit of the Duke Regional Biocontainment Laboratory (RBL).

### Analysis of The Cancer Genome Atlas (TCGA) data

All TCGA data was downloaded from https://tcga-data.nci.nih.gov/docs/publications/tcga/ and through cbioportal.org^82,83^. The most recently published 2013 GBM data set was used for all analyses. For each analysis, the maximum number of complete cases available (confirmed *IDH1/2* wildtype) were used unless otherwise stated, as IDH mutations are known to independently influence epigenetics and cellular differentiation. Analysis was carried out using Graphpad Prism, Microsoft excel, Genesis, and Statgraphics software. To perform this analysis, samples were ordered by MTAP expression level or equally divided into quartiles based on MTAP expression and statistical comparisons were made between the “low” and “high” expression groups to identify genes that may be impacted by *MTAP* status. Benjamini–Hochberg correction was performed to account for multiple testing error. We utilized *MTAP* expression levels to categorize patients rather than gene copy number because *MTAP* is known to be silenced epigenetically in a variety of cancer types^84–87^, and our analysis of DNA methylation and gene expression data in patients suggests it can also be epigenetically silenced in GBM^21^.

### Statistical analysis

Statistical tests (student’s t test, ANOVA) were performed using Graphpad Prism. All experiments were repeated to ensure reproducibility of results. Unless otherwise indicated, pooled data from multiple experiments was used for each figure. A *P* value cutoff of 0.05 was used to determine significance in all cases except where corrections were applied for larger data sets (i.e., Benjamini-Hochberg, Bonferonni).

## Supplementary Information

Supplementary Information 1.

Supplementary Information 2.

Supplementary Information 3.
