# Supplementary material for: MTAP loss correlates with an immunosuppressive profile in GBM and its substrate MTA stimulates alternative macrophage polarization
Source: Sci Rep. 2022 Mar 9;12:4183. doi: 10.1038/s41598-022-07697-0 (PMC8907307; doi:10.1038/s41598-022-07697-0)
Supplement: Supplementary file 1 — Supplementary Information 1. [file 41598_2022_7697_MOESM1_ESM.docx]

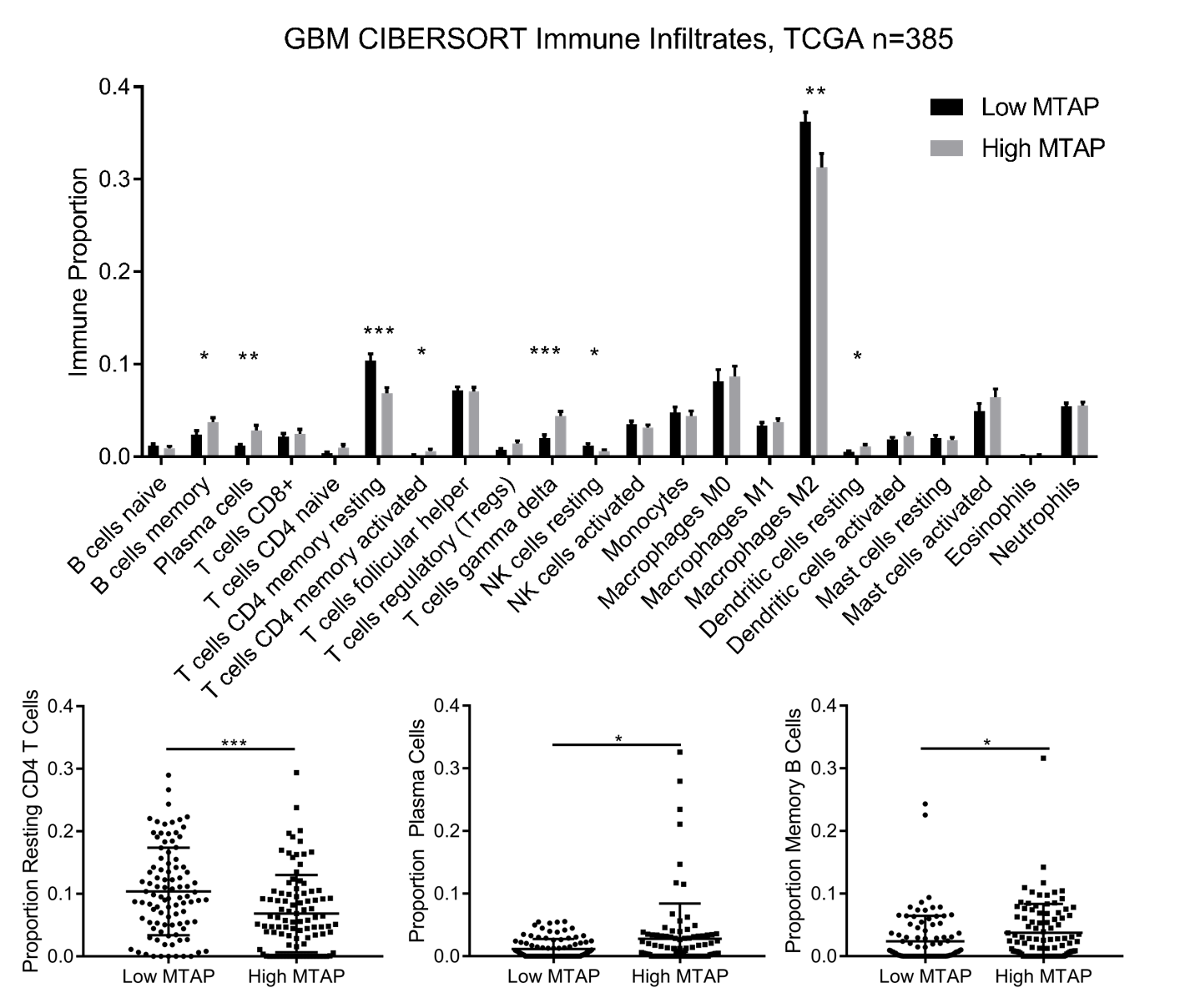


**Supplementary Fig. S1**. CIBERSORT analysis on TCGA GBM microarray gene expression data reveals several immune cell types which are differentially represented between samples with low *MTAP* expression and samples with high *MTAP* expression. Bottom graphs show individual data points for resting CD4 T cells, plasma cells and memory B cells. See Figure 1 for graphs of Activated CD4 T cells, M2 Macrophages and gamma delta T cells. Comparison was made between upper and lower quartiles, n=96, mean +/- standard deviation, t test *P* value * *P* < 0.05, ** *P* < 0.005, *** *P* < 0.0005.

**
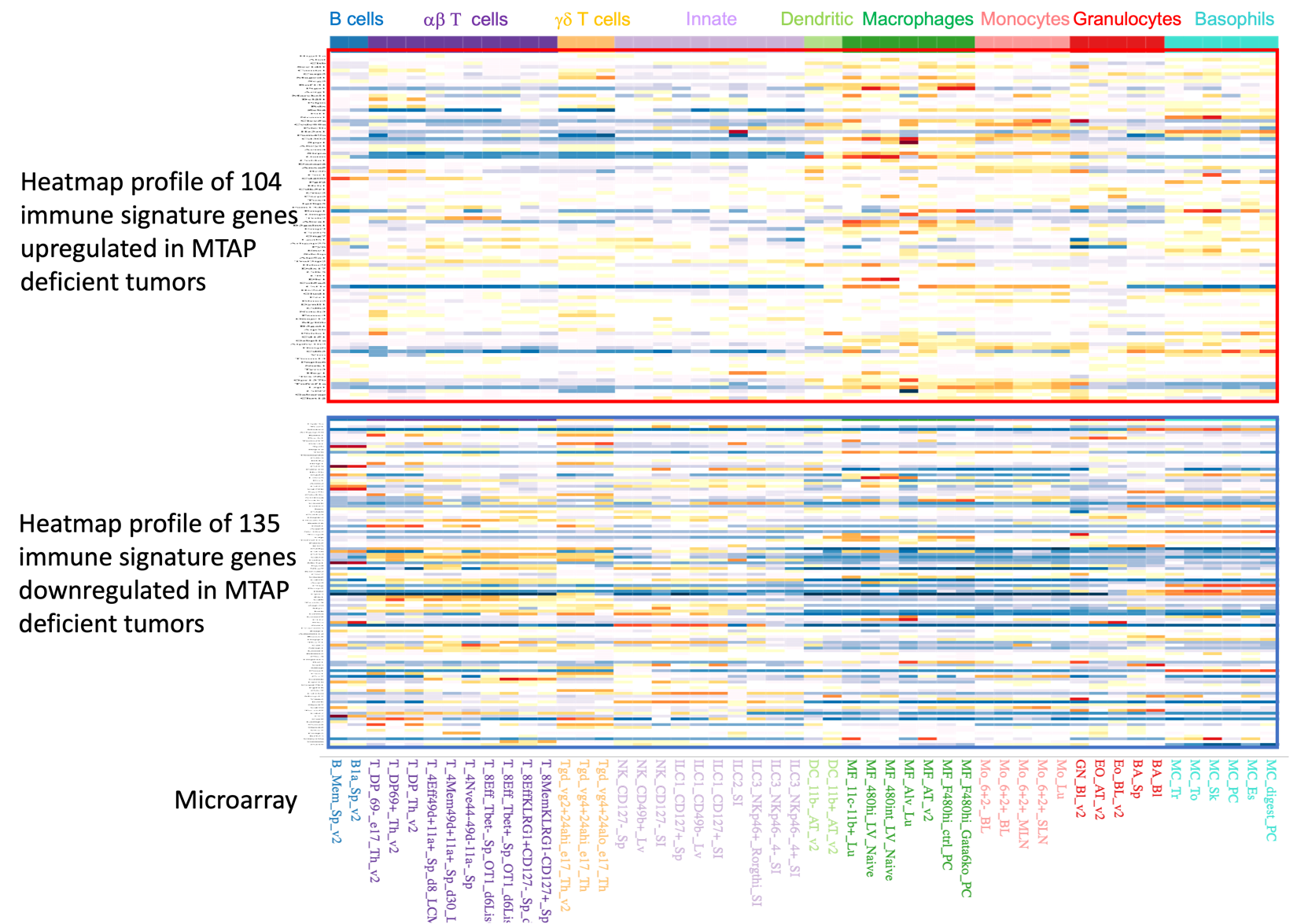
Supplementary Fig. S2.** Enlarged heatmaps from gene expression analysis shown in Figure 1D. Microarray data is from a compendium of gene expression datasets (listed on x axis) representing various immune cell types available listed along the top. Database available at https://www.ImmGen.org. The list of genes queried (y axis, see supplementary tables) was generated by identifying immune cell marker genes that are differentially expressed between samples with low or high *MTAP* expression in TCGA GBM microarray data. The top half of the figure represents immune signature genes upregulated in MTAP deficient GBM, with a profile that favors macrophages, monocytes, basophils, dendritic cells. The bottom half of the figure represents immune signature genes downregulated in MTAP deficient GBM, composed primarily of T cells and innate lymphocytes.


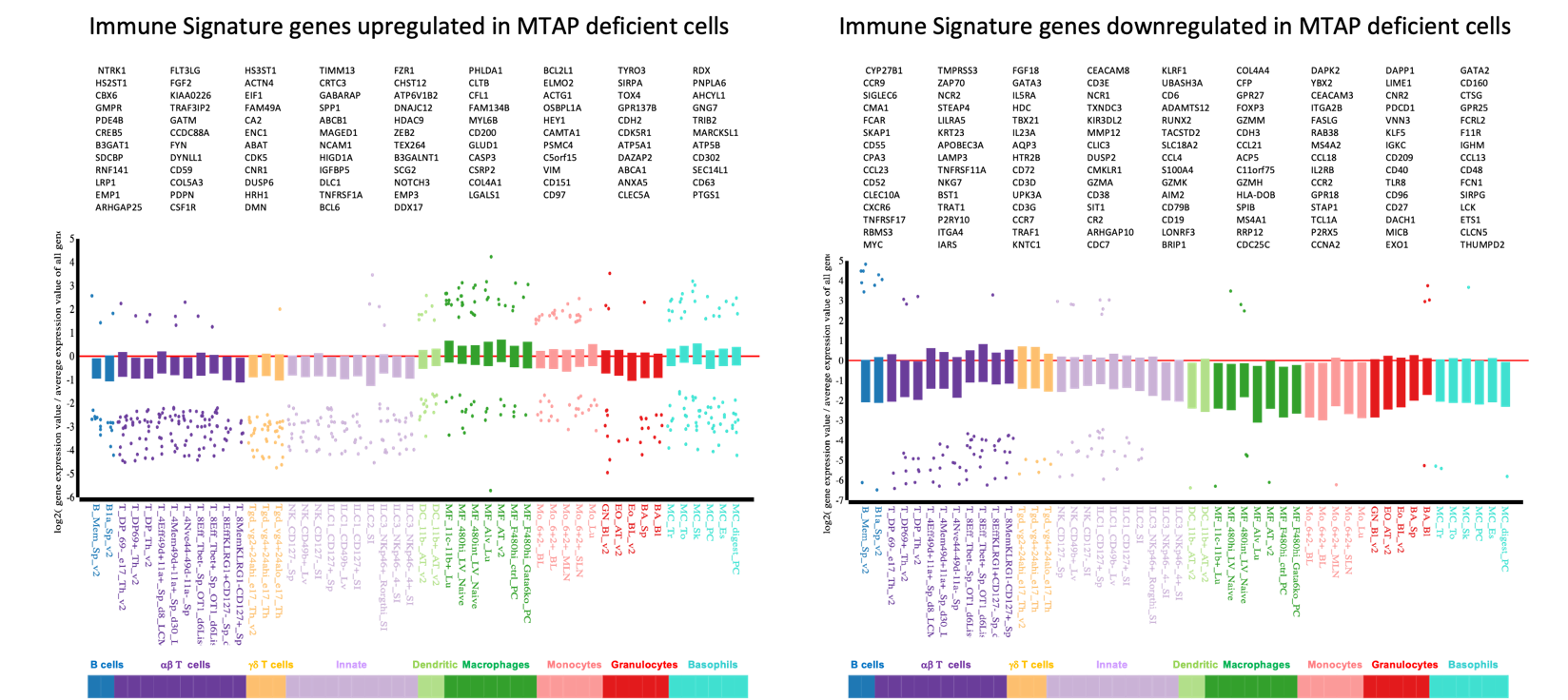


**Supplementary Fig. S3.** Gene lists and w plots showing immune signature genes represented in Figure 1D and Supplementary Figure 2S. There are 104 immune signature genes upregulated in MTAP deficient samples and 135 immune signature genes downregulated in MTAP deficient samples, Benjamini-Hochberg corrected p<0.0182. W Plots illustrate the expression levels of the displayed gene set in the various immune cell types represented, based on compiled microarray data, with the 50 microarray studies illustrated across the x axis. The y axis shows gene expression value of the listed genes compared to average of all other genes. Immune signature microarray database is available at https://www.ImmGen.org.

**
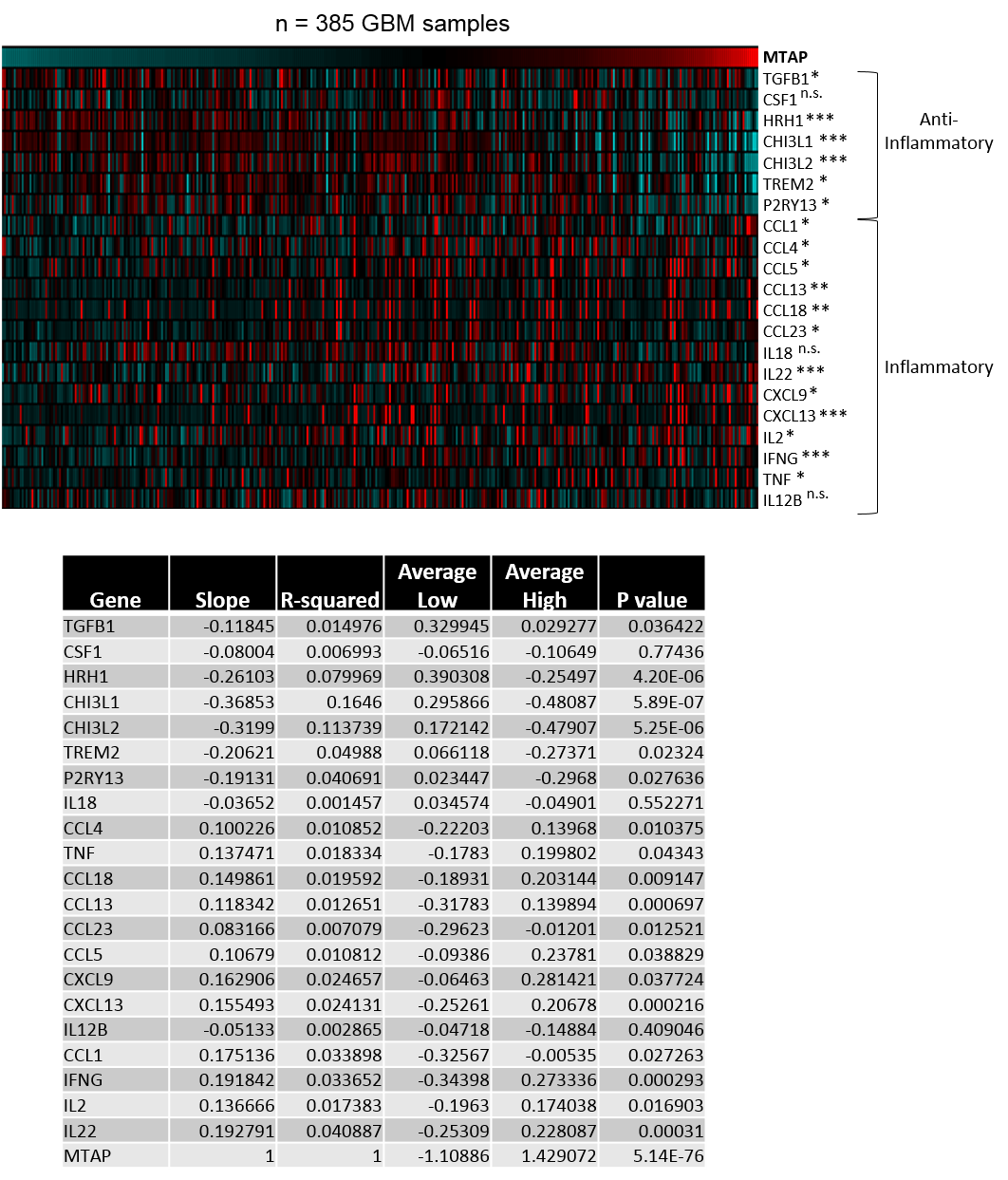
**

**
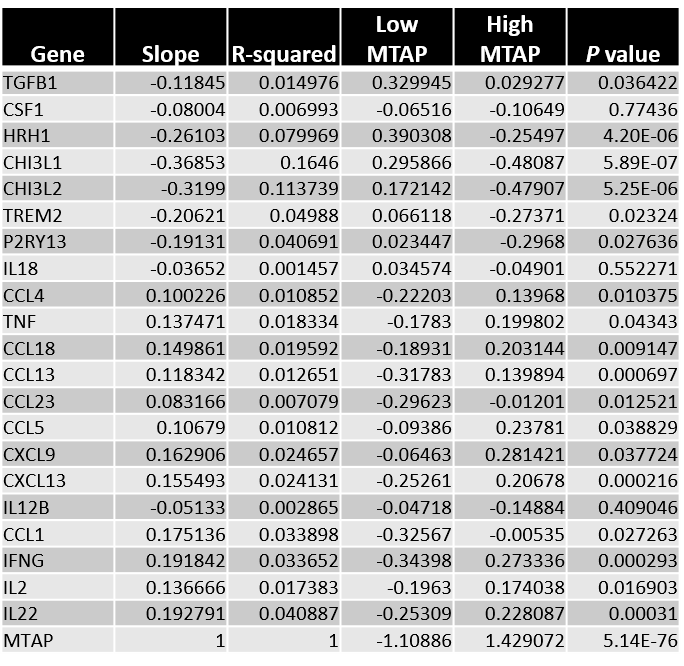
**

**Supplementary Fig. S4 .** Top: Heatmap showing expression correlation of selected immune-related genes with *MTAP* expression (top row) from the TCGA GBM dataset. Table on bottom indicates characteristics of the regression of each gene compared to *MTAP* expression (gene expression = *MTAP* expression*slope + y-intercept). The average expression level of each gene in the lowest MTAP expression quartile (low MTAP) and the highest MTAP expression quartile (high MTAP) are shown. The last table column indicates the *P* value from a t test comparing the expression of each gene within the low MTAP quartile with the expression of that gene in the high *MTAP* quartile.


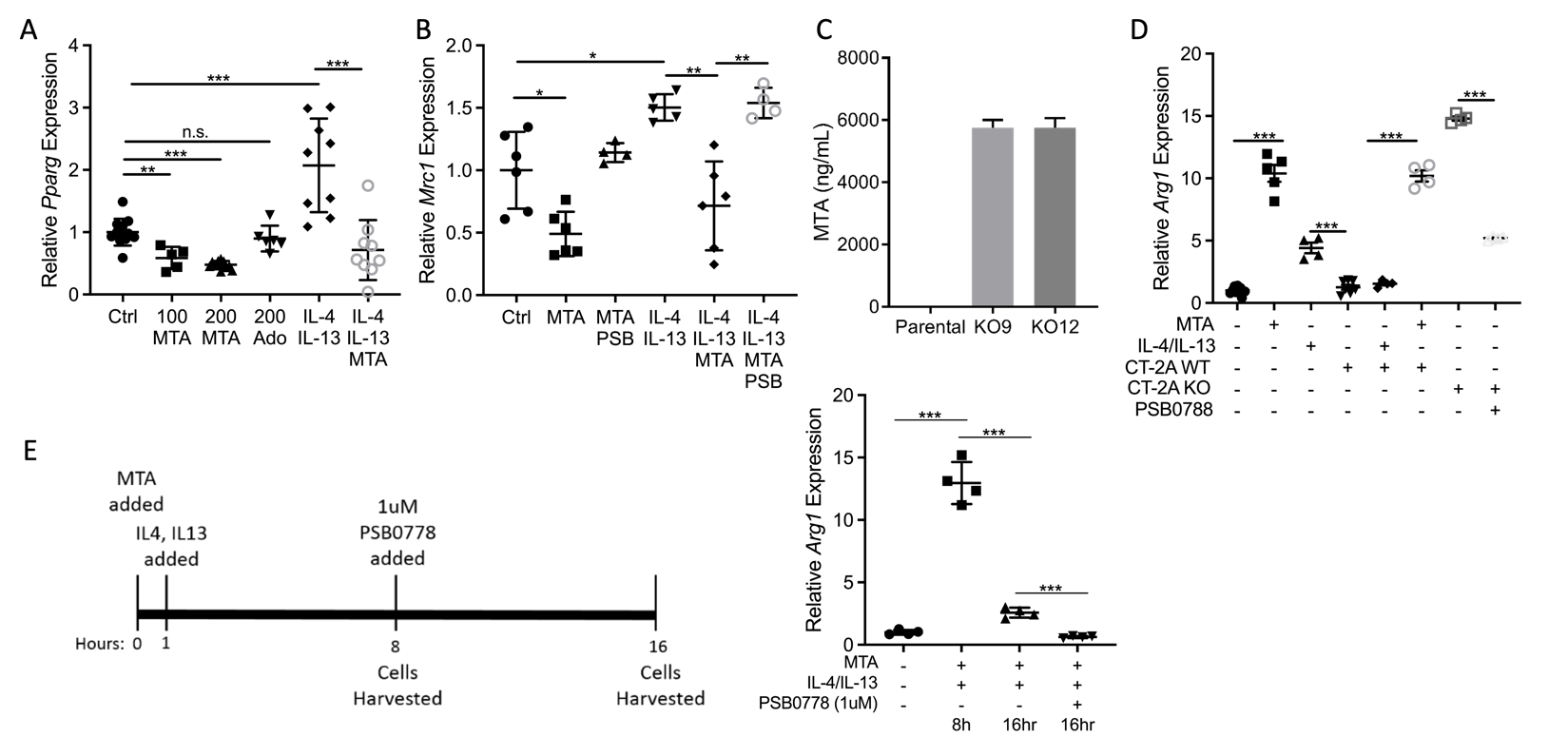


**Supplementary Fig. S5.** **(A)** RAW 264.7 cells were treated with indicated cytokines for 12 hours and total RNA was isolated for measuring PPARG expression, (ado=adenosine), n ≥ 5. **(B)** RAW 264.7 cells were treated with the indicated cytokines with or without 100µM MTA for 12 hours, and total RNA was isolated for measuring expression of *Mrc1,* (PSB = A_2B_R inhibitor, PSB0778), n ≥ 5. (**C**) MTA was measured in spent cell culture media from CT-2A parental and two *MTAP* knockout cell lines (#9 and #12) using liquid chromatography tandem mass spectrometry (LC-MS/MS), n=2. (**D**) Raw 264.7 cells were exposed to media collected from parental or *Mtap* knockout CT-2A cells for 12 hours before *Arg1* gene expression was measured by RT-qPCR, n ≥ 4. **(E)** Left: timeline for delayed administration of A_2B_R inhibitor PSB0788 following MTA treatment for data shown on right. *Arg1* expression was measured by RT-qPCR. All experiments were repeated independently. All statistical comparisons were performed using an ANOVA for group comparisons or unpaired student’s t-test for individual comparisons; error bars indicate mean +/- SD; * = *P*<0.05, ** = *P*<0.005, *** = *P*< 5x10^-4^, n.s. = not significant.


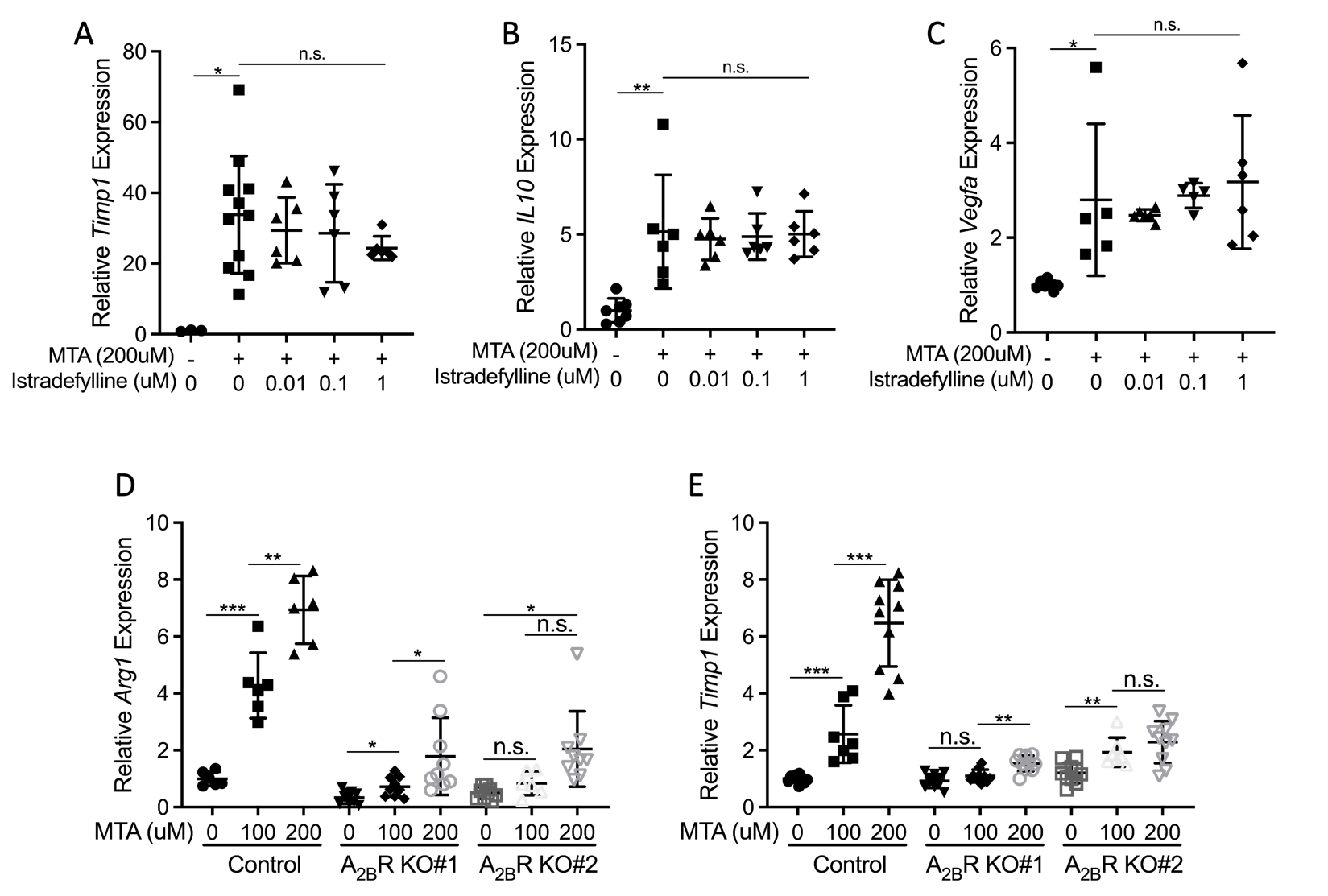


**Supplementary Fig. S6.** **(A-C)** Raw 264.7 cells were treated with MTA and varying doses of A_2A_ receptor inhibitor istradefylline and **(A)** *Timp1*, **(B)** *IL10*, and **(C)** *Vegfa* expression were measured by RT-PCR, n ≥ 5. (**D**, **E**) The A_2B_ receptor was knocked out using CRISPR-Cas9. Two separate knockout populations were tested along with an empty vector control. Cells were treated for 12 hours with MTA and **(D)** *Arg1* and **(E)** *Timp1* expression were measured by RT-PCR, n ≥ 5. All statistical comparisons were performed using ANOVA for group comparisons or an unpaired student’s t-test for individual comparisons; error bars indicate mean +/- standard deviation; * = *P*<0.05, ** = *P*<0.005, *** = *P*< 5x10^-4^, n.s. = not significant.


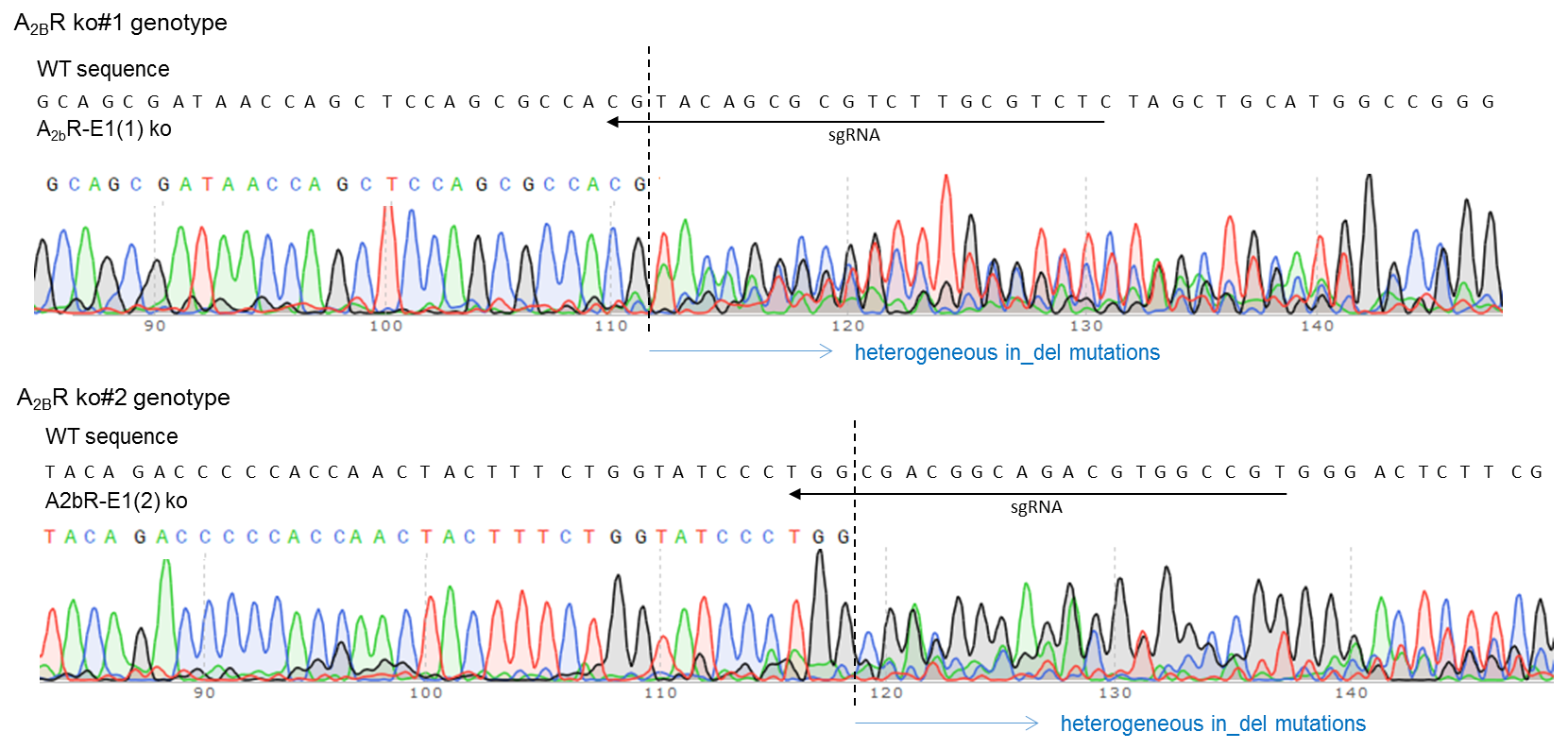


**Supplementary Fig. S7.** Genotyping of the A_2B_R exon 1 targeted by sgRNA#1 (ko#1) and sgRNA#2 (ko#2). sgRNA sequences used are underlined and expected cut sizes are marked. Note the heterogeneous in-del mutations following the expected sgRNA cut sites.


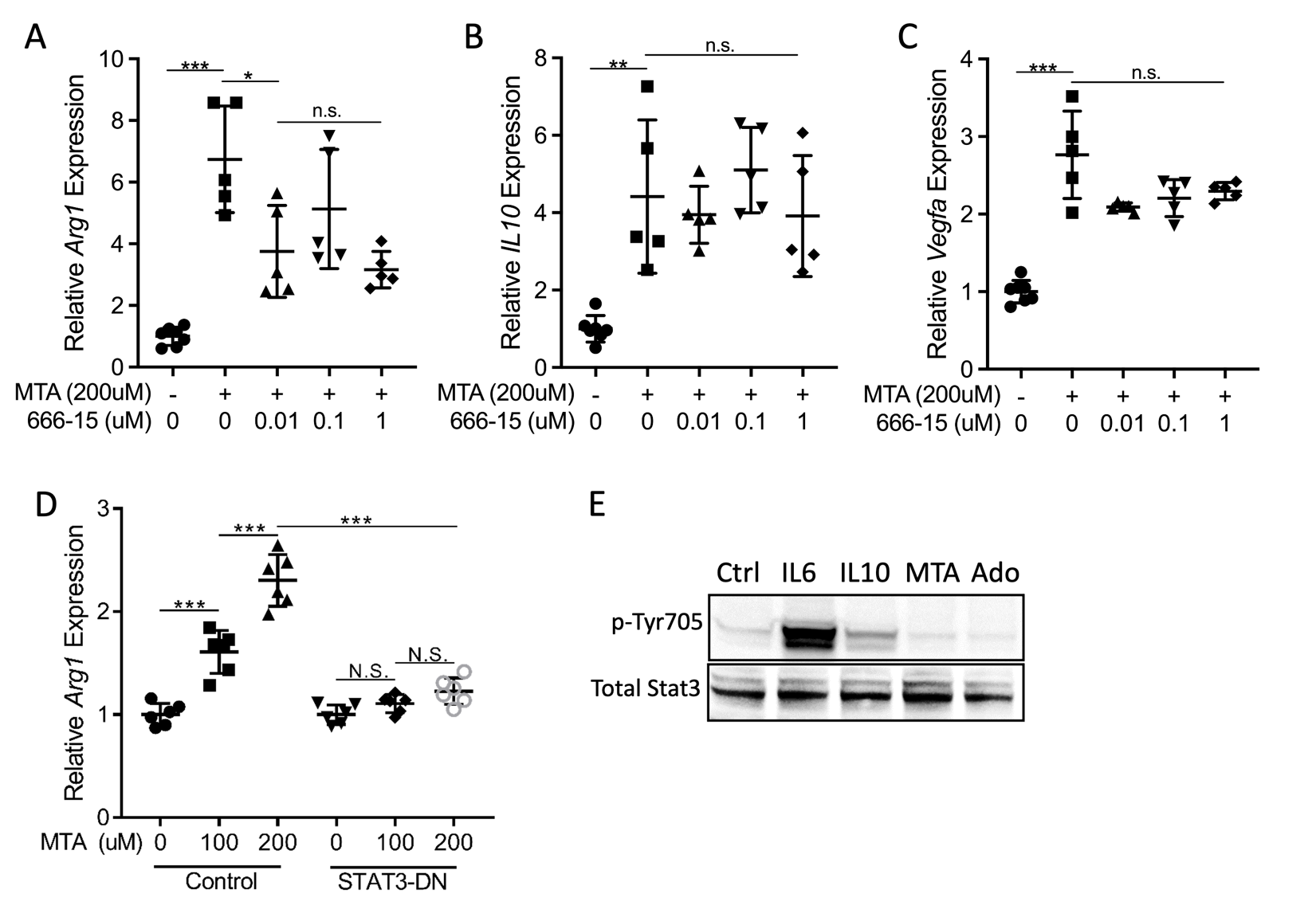


**Supplementary Fig. S8.** **(A-C)** RAW264.7 cells were treated with MTA and varying doses of CREB inhibitor 666-15 for 12 hours and **(A)** *Arg1*, **(B)** *IL10*, and **(C)** *Vegfa* expression were measured by RT-PCR. **(D)** A dominant-negative form of STAT3 (STAT3-DN) or empty vector control were expressed in RAW 264.7 cells. Cells were treated with MTA for 12 hours and *Arg1* expression was measured by RT-PCR. **(E)** RAW 264.7 cells were treated with MTA, Adenosine (Ado), and positive controls IL-6 and IL-10 for 1 hr then harvested and western blot was done to measure phospho-STAT3 (Tyr705) and total STAT3 protein levels. All statistical comparisons were performed using ANOVA for group comparisons or an unpaired student’s t-test for individual comparisons. Error bars indicate mean +/- standard deviation; * = *P*<0.05, ** = *P*<0.005, *** = *P*< 5x10^-4^, n.s. = not significant.


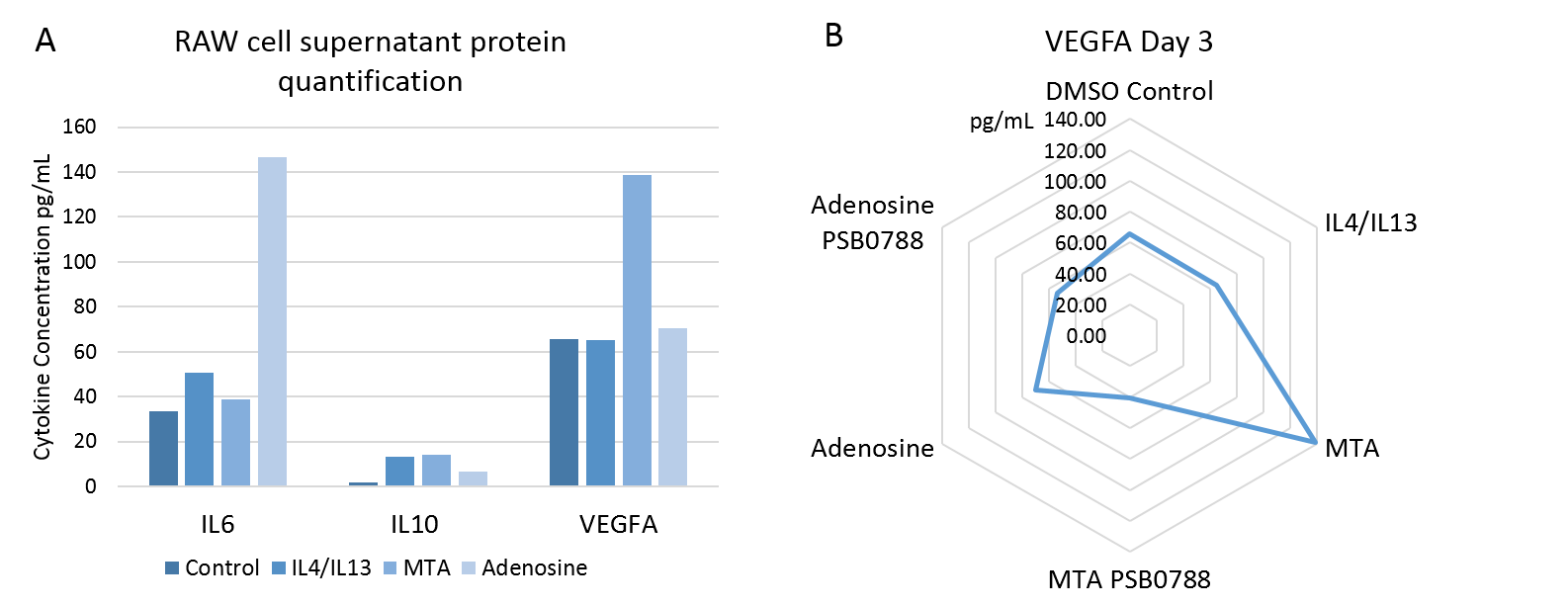


**Supplementary Fig. S9.** RAW 264.7 cells were treated with MTA, Adenosine, and IL4/IL13 and supernatants were collected after 3 days and measured by Milliplex. **(A)** IL-6, IL-10, and VEGFA quantification. **(B)** VEGFA concentration is shown for each of 6 conditions, with MTA producing the strongest induction of VEGFA cytokine production.
