# Supplementary figures and images for: MTAP loss correlates with an immunosuppressive profile in GBM and its substrate MTA stimulates alternative macrophage polarization
Source: Sci Rep. 2022 Mar 9;12:4183. doi: 10.1038/s41598-022-07697-0 (PMC8907307; doi:10.1038/s41598-022-07697-0)

**Supplementary Infomration:** Whole gel images for immunoblots


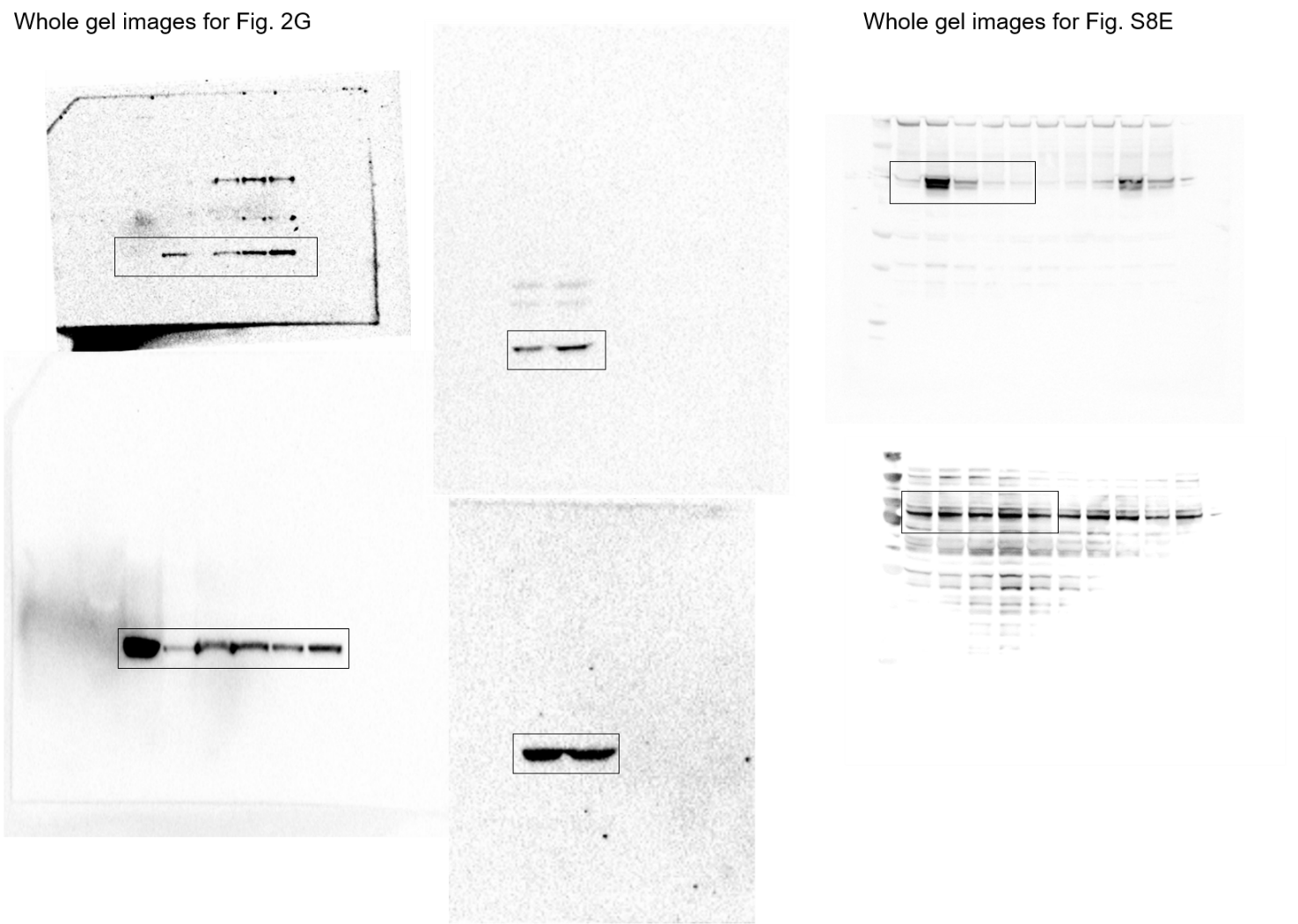

Supplement: Supplementary file 2 — Supplementary Information 2. [file 41598_2022_7697_MOESM2_ESM.docx]
